# Radiation exposure during orthodontic treatment: risk to children and adolescents

**DOI:** 10.2340/aos.v83.40571

**Published:** 2024-05-15

**Authors:** Christina Stervik, Agneta Lith, Annika Ekestubbe

**Affiliations:** Department of Oral and Maxillofacial Radiology, Institute of Odontology, Sahlgrenska Academy at University of Gothenburg, Gothenburg, Sweden

**Keywords:** dental radiography, indication, orthodontic treatment, panoramic, radiation risk

## Abstract

**Objectives:**

To estimate radiation risk to children and adolescents during orthodontic treatment by retrieving number and type of radiographs from the patient records.

**Material and methods:**

Radiographs, along with justifications for radiation exposure, were obtained retrospectively from the patient records of 1,790 children and adolescents referred to two Swedish orthodontic clinics. Data were grouped according to treatment stage: treatment planning, treatment, and follow-up. Estimated risk was calculated using the concept of effective dose.

**Results:**

Each patient had received around seven radiographs for orthodontic purposes. The most common exposures during treatment planning were one panoramic, one lateral, and three intraoral periapical radiographs. A small number of patients received a tomographic examination (8.2%). Few justifications for treatment planning and follow-up, but more in the actual treatment stage, had been recorded. The most common examinations were to assess root resorption and the positions of unerupted teeth, or simply carry out an unspecified control. The estimated risk of developing fatal cancer was considered low. The radiation risk from orthodontic treatment was equivalent to about 5–10 days of natural background radiation.

**Conclusions:**

Children and adolescents sometimes undergo multiple radiographic examinations, but despite the low radiation burden, accumulated radiation exposure should be considered and justified in young patients.

## Introduction

In developed countries, orthodontic treatment is a common procedure for correcting a malocclusion of the deciduous and permanent teeth. Treatment usually begins at 12–14 years of age after all permanent teeth have erupted. At this age, the patient is also considered to possess a sense of autonomy in being able to accept or reject orthodontic treatment [1].

In the four Nordic countries, between 11% and 35% of children and adolescents receive orthodontic treatment; however, variations between areas and countries are large [2]. In Sweden, nearly every fourth child or adolescent – approximately 400,000 of the 6–19-year-olds – receives some kind of orthodontic treatment [1, 3]. Patients considering orthodontic treatment will first undergo a clinical examination to inform diagnosis and therapy planning. As a diagnostic aid, radiography will often supplement the examination and is considered valuable for diagnostic and treatment decisions [4]. Bruks et al. [5], however, found that radiographs are not always necessary for treatment planning; in most cases, a clinical examination, study casts, and photographs provide adequate information. Despite these findings, a recent questionnaire study found that nearly all orthodontists take radiographs, especially during treatment planning and treatment [6].

Radiation risk is age dependent: younger individuals are more sensitive than adults [7]. Unlike adults, children are still growing and thus have more tissue undergoing rapid cell growth; their expected remaining lifetime is also longer. Furthermore, healthy children and adolescents rarely have oral diseases, which can be relevant and a reason for radiographic examination in older individuals [8].

Before any radiographic examination, a clinical examination must be done in order to avoid unnecessary exposure to radiation [7, 9]. To avoid radiographs being taken simply because it is ‘routine’, the justification should conclude that the needed information is not available elsewhere and that radiography is the most suitable method for obtaining the information [9]. The Swedish National Board of Health and Welfare has developed evidence-based guidelines for both adult and child dental care for promoting equity in oral health and dental care. The guidelines are an aid for clinicians in their treatment decision [10]. However, no such guidelines for children and adolescent concerning orthodontic treatment are yet available.

Thus, as a step in this direction, the present study estimates radiation risk to children and adolescents during orthodontic treatment by retrieving number and type of radiographs from the patient records.

## Subjects and methods

The present study is retrospective and includes 1,790 children and adolescents who completed orthodontic treatment at two clinics (A and B). Data collection occurred between 2004 and 2005 at clinic A, and between 2011 and 2012 at clinic B. Table 1 summarises the characteristics of the study population. Both clinics were located within a metropolitan catchment area in the same county council. Both employed specialist-trained orthodontists and offered specialist treatment; clinic A also offered post-graduate education.

**Table 1:** Descriptive characteristics of the study cohort.

	Patients (*n*)	Age years (mean ± SD)	Sex	
			Female	Male
**Total**	1,790	14.8 ± 2.57	1,063	727
**Clinic A**	1,125	14.1 ± 2.45	652	473
**Clinic B**	665	16.0 ± 2.37	411	254

Patients with a cleft palate and other craniofacial disorders were excluded from the study. Other exclusion criteria were incomplete patient records, incomplete treatment, and moving from the area.

### Clinical data

Number and type of radiograph taken in conjunction with orthodontic treatment at the two clinics were noted, as well as their justifications and when they were taken, whether during (1) treatment planning, (2) treatment, or (3) follow-up. For patients who had needed supplemental radiographs and been referred to specialist clinics in oral radiology, the justifications were retrieved from the referral document.

### Effective dose and risk calculation

The stochastic risk of inducing fatal cancer is associated with the effective dose. Effective dose, as reported in Granlund et al. [11], Ludlow et al. [12], and Radiation Protection No. 172 [13] was used to calculate the stochastic risk of the various dental examinations (Table 2). The risk factor for fatal cancer was calculated as 15% per Sievert (Sv) for children under 10 years and 10% per Sv for patients between the age of 10 and 20 years [14]. Risk estimations for the present study cohort were made for each age group.

**Table 2:** Effective doses for various radiographic techniques.

Radiographic examination	Effective dose (*µ*Sv/image)
Panoramic radiograph	14.2*–36**
Lateral cephalogram	5.6*
Intraoral (single)	0.8**
*CBCT dento-alveolar*	
10-year-old	43***
Adolescent	32***

* Ludlow et al. 2008;

** Granlund et al., 2016;

*** Radiation protection No. 172; CBCT: cone-beam computed tomography.

The Regional Ethics Review Board in Gothenburg, Sweden, granted ethics approval for the present study ([Dnr] 380–11).

### Data analysis

Statistical analyses were done using the *t*-test for comparison between the two groups. A *p* < 0.05 was considered significant.

## Results

Table 3 presents the distribution and number of radiographs taken during the three treatment stages and in the two age groups from clinics A and B. Of the 1,790 children and adolescents, mere 1% (20) entered treatment without radiographs.

**Table 3:** Number of radiographs by age group, radiographic technique, and treatment stage.

Age groups (years)	Patients (*n*)	Treatment planning			Treatment			Follow-up		
		Pan	Lat	IO	Pan	Lat	IO	Pan	Lat	IO
**All**										
< 10	38	42	27	64	12	9	32			
10–20	1,752	1,864	1,613	5,133	328	240	4,386	50	26	480
**Clinic A**										
< 10	28	32	18	38	12	9	32			
10–20	1,097	1,156	940	2,474	287	213	2,690	42	26	25
**Clinic B**										
< 10	10	10	9	26						
10–20	655	708	673	2,659	41	27	1,696	8		455

Pan: panoramic radiography; Lat: lateral cephalogram; IO: intraoral periapical radiography.

**Figure 1:**
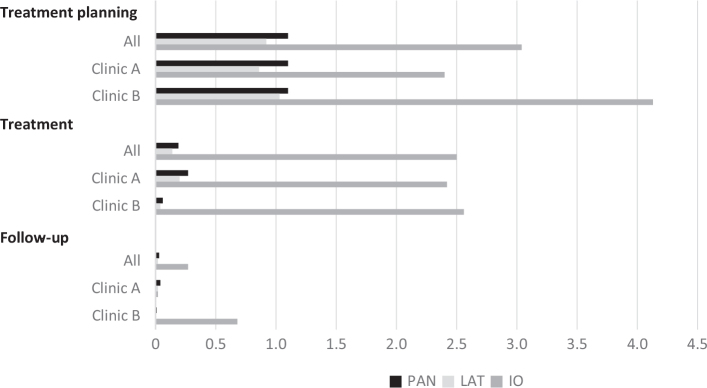
Number of exposed radiographs per individual (nstudy cohort = 1,790) during orthodontic treatment by stage (treatment planning, treatment, and follow-up) and clinic (A and B).

At the treatment planning stage, the most common radiographic examination was panoramic radiography, together with a lateral cephalogram and approximately three intraoral periapical radiographs. Clinic B took more intraoral radiographs (IO) per individual than clinic A during treatment planning (the mean number of IO/patient were 4.0 and 2.2 in clinic B and A, respectively; *p* < 0.001), and follow-up (the mean number of IO/patient were 0.7 and 0.02 in clinic B and A, respectively; *p* < 0.001). Figure 1 shows the number of radiographs per individual in each of the three stages.

Table 4 presents the supplementary radiographs taken after referral to an oral radiology clinic. In the study cohort, 8.2% (146) of the patients received a tomographic examination. Cone-beam computed tomography (CBCT) with a 4 × 4 cm field of view (FOV) was used to examine 110 (6.1%) of the patients, and a 6 × 6 cm FOV, was used for 20 (1.1%) of the patients. Five patients were examined with computed tomography (CT) and 11, with conventional motion tomography.

**Table 4:** Number of supplementary radiographs taken on referral to an oral radiology department by age group and radiographic technique.

Age group (years)	Patients (n)	Supplementary radiographs (*n*)		
		Tomography*	Extraoral radiography**	Intraoral radiography***
**All**				
<10	38	2		2
10–20	1,752	144	50	238
**Clinic A**				
<10	28	2		2
10–20	1,097	101	44	179
**Clinic B**				
<10	10			
10–20	655	43	6	59

CBCT: cone-beam computed tomography; CT: computed tomography.

* Conventional motion tomography.

** Panoramic radiography, scanogram, lateral cephalogram.

*** Periapical, occlusal radiography.

The number of radiographic examinations with justifications noted in the patient histories varied for each stage, being more frequent during the treatment stage than the planning and follow-up stages (Table 5). Table 6 presents the reasons for the examination. The most common were simply to check (unspecified, 48%) and to determine root resorption (39%), or the position of unerupted teeth (9%). Retakes were 2%. All justifications for the supplementary radiographs were clearly specified on the referrals.

**Table 5:** Number of radiographs with justifications as recorded in the patient history, by stage.

Justifications	Planning (*n* = 1,770)	Treatment (*n* = 1,006)	Follow-up (*n* = 187)
*n*	85	606	28
%	5	60	15

**Table 6:** Types of justifications recorded in the patient histories.

Justification
Presence/absence of permanent teeth
Position of unerupted teeth
Teeth inclination
Root anatomy
Intermaxillary relationship
Trauma control
Marginal bone loss
Root resorption
Apical disease
Cyst-like lesion
Deviation in form of mandibular condyle
Control
Retake

Table 7 presents the estimated risk of fatal cancer by age group and radiographic technique. For example, a group of young subjects under 10 years of age who received one panoramic radiograph would have a theoretical risk of 0.54 radiation-induced fatal cancers per 100,000 irradiated individuals; individuals between 10 and 20 years of age would have a theoretical risk of only 0.36 per 100,000 irradiated individuals.

**Table 7:** Risk of fatal cancer to children and adolescents by age group and radiographic technique.

Radiographic technique	Effective Dose (*µ*Sv)/image	Risk of fatal cancer (%/mSv)	Collective dose per 100,000 persons (man-mSv)	Induced fatal cancer per 100,000 persons (*n*)
**<10 years**				
Panoramic	14.2*	0.015	1,420	0.21
Panoramic	36**	0.015	3,600	0.54
Lateral	5.6*	0.015	560	0.10
Intraoral	0.8**	0.015	80	0.01
CBCT	43***	0.015	4,300	0.65
**10–20 years**				
Panoramic	14.2*	0.01	1,420	0.14
Panoramic	36**	0.01	3,600	0.36
Lateral	5.6*	0.01	560	0.06
Intraoral	0.8**	0.01	80	0.01
CBCT	32***	0.01	3,200	0.32

CBCT: cone-beam computed tomography.

* Ludlow et al. 2008;

** Granlund et al. 2016;

*** Radiation protection No. 172.

## Discussion

In the study cohort 14,306 radiographs or approximately seven radiographs per individual were exposed for purposes of orthodontic treatment. Thus, in Sweden, around 2.8 million radiographs (panoramic, lateral, and intraoral) are exposed during orthodontic treatment of 400,000 individuals. The Swedish government finances orthodontic treatment based on objective needs. The proportion of children and adolescents in treatment would probably increase above the current 25% of that age group if the government funded treatment for aesthetic reasons.

Young people are more sensitive to radiation than adults due to a higher cell turnover and a longer remaining life expectancy, which exceeds the latent period between X-ray exposure and the emergence of a tumour. Therefore, it is important to investigate the use of X-rays in dentistry, for example in connection with orthodontic treatment, because there is a possible theoretical connection between medical X-ray examinations during childhood and adolescence, and the risk of developing cancer [15].

The present study estimated the risk of fatal cancer later in life from one panoramic radiograph; using the effective dose concept, that risk was 0.5 per 100,000 children and adolescents, indicating a probability of two fatal cancers in a population of 400,000 individuals. Besides a high cell turnover and longer remaining life expectancy, the organs of children and adolescents are closer together due to a smaller body diameter than adults; thus, the radiation risk may be even higher for this age group since risk calculations based on effective doses are determined for adults [16].

To minimise radiation-induced risks for the individual as well as larger populations, each radiation exposure must be justified and optimised. The recommended principle, ‘As Low As Reasonably Achievable’ (ALARA) should be applied on a daily basis. The benefit of the additional information must always be weighed against the risks attached to the use of ionising radiation [9]. The present study found that documented indications varied between 5% and 60% in the treatment stages. Our findings agree with Svenson et al. [17], who reported that 64% of Swedish dentists responding to a questionnaire on choice of digital radiography stated that they made bitewing radiographs for each new patient, and that 75% of these exposures had no indications. Svenson et al. also observed that dentists with longer clinical experience were less likely to use individual indications. Our study did not query the clinical experience of the orthodontists therefor, associations between clinical experience and individual justifications were un-assessable.

The lowest percent of recorded justifications per exposure occurred in the treatment planning stage: 5% (85) of 1,790 examinations. The fact that the indications are common and obvious may be one reason why no justification was recorded. An administrative system that fails to require documentation of radiographic examinations or their justifications may be another reason. However, during the treatment phase, justifications were recorded for 60% of the examinations, indicating that radiographs are taken for a specific reason.

The Swedish Radiation Safety Authority in 2022 published a report regarding the justification of radiographs used in child and adolescent dentistry [18]. Conclusions drawn in this report were that there is potential for improvement in clarifying the justifications process so that it includes all X-rays modalities and roles of responsibility within both general dentistry and specialist dentistry.

A panoramic exposure was the radiograph most commonly taken, predominantly during treatment planning, which is in line with a study on the radiographic preferences of orthodontists for orthodontic treatment [6]. Few justifications for panoramic radiography were recorded in the patient histories. A panoramic radiograph, however, may be the best choice, even if individual indications are lacking. In a previous questionnaire study, orthodontists reported various reasons for taking panoramic radiographs, the most common being the wish for an overview of the jaws and for an alternative to IO to determine the presence or absence of permanent teeth [6]. Another study, on errors and pathology in panoramic images of orthodontic patients, showed that hypodontia and impacted teeth were common findings in tooth bearing regions [8]. However, pathological changes outside the tooth bearing regions, that would require panoramic radiography, were found in 13 (1%) out of 1,287 patients. The impact of pathological findings must be weighed against the increased radiation risk associated with panoramic radiography in healthy children and adolescents.

One limitation of the present study was that no assessment of the benefit of the radiographs was done concerning treatment outcome. However, other, less dose demanding technologies such as bitewing radiographs can instead be used, since orthodontic treatment is usually begun when most or all permanent teeth have erupted. The benefit of the intraoral technique is high due to low radiation dose and low cost [19]. On the other hand, when the patient cannot tolerate an intraoral detector, extraoral radiography is preferable, particularly for third molar assessment. To evaluate root resorption, which was the most common indication for intraoral radiography in the present study, IO are recommended over panoramic radiographs [20]. During the treatment planning stage in the present study, one lateral cephalogram besides a panoramic was exposed for almost all children and adolescents. One systematic review, however, concluded that lateral cephalograms made no significant difference in treatment planning decisions [21], while another study found that lateral cephalograms had a high impact on treatment planning [5].

CBCT or CT was used as a supplement in more complicated cases, such as impacted teeth or resorption of an adjacent tooth. A small FOV was used in 85% of the cases, which is in line with recommendations [13]. Both CT and CBCT could be used to supplement conventional radiography, but only in select cases due to the high radiation dose. Our study showed that tomography, although sparsely used, was optimised according to European Commission recommendations.

Differences between the clinics in use of radiographic procedures were minor, even though one clinic (A) also offered specialist training. Clinic B did use intraoral periapical radiographs more frequently. The data from clinic B is 7–8 years later in time, which may explain this difference since knowledge of side effects such as root resorption and marginal bone loss in connection with treatment had increased during this interval [22, 23].

It is well known that exposure to high radiation doses can affect health, and doses below 100 milligray (mGy) are in the low-dose range where mainly stochastic effects occur. The linear non-threshold model is used in low-dose ranges to estimate stochastic effects; it assumes that there is no threshold dose below which there is no additional health risk. According to the model, risk increases linearly with absorbed dose [24]. Radiation doses in dental radiographic examinations are in the low-dose range, and compared with background radiation, the dose is negligible. Annual exposure from natural background radiation in Sweden has been calculated as 1–2 mSv [25]; thus, the additional radiation dose to children and adolescent in orthodontic treatment corresponds to 5–10 days of background radiation. Furthermore, a panoramic examination delivers a dose that is six times higher than a lateral cephalogram and 4–5 times higher than an intraoral image. Until there is clear evidence for a threshold dose below 100 mGy, we must assume that radiography involves a small albeit real risk to the patient. The difficulties are in estimating the risk of low-risk-level exposures, such as occurring in dentistry [12]. In 2007, the International Commission on Radiological Protection (ICRP) revised estimates of the radiosensitivity of tissues and organs, and this has resulted in reassessments of earlier estimations of risk from dental radiographic examinations. This is relevant for orthodontic treatment, which often entails many exposures for children and adolescents.

For the individual, dental radiography is associated with low doses and risks; however, on the population level, it is considered a ‘high-volume procedure’ [7]. Although radiation risk is considered to be low, children may need to undergo many radiological procedures, dental or otherwise, early in life. Thus, accumulated radiation exposure should be considered when scheduling children and adolescents for radiographic examinations. Clinicians should always justify the need for a radiographic examination, citing how the benefits outweigh the potential harm.
